# Involvement of aryl hydrocarbon receptor signaling in the development of small cell lung cancer induced by HPV E6/E7 oncoproteins

**DOI:** 10.1186/1479-5876-9-2

**Published:** 2011-01-04

**Authors:** Tonia Buonomo, Laura Carraresi, Mara Rossini, Rosanna Martinelli

**Affiliations:** 1 CEINGE Biotecnologie Avanzate, Via Comunale Margherita 482, 80145 Napoli, Italy; 2 Metabolic and Muscular Unit, Clinic of Paediatric Neurology, A.O.U Meyer, Viale Pieraccini 6, 50139 Florence, Italy; 3 Department of Physiopathology, Experimental Medicine and Public Health, University of Siena, 53100 Siena, Italy; 4 Department of Biochemistry and Medical Biotechnologies, University of Naples, "Federico II", 80131 Naples, Italy

## Abstract

**Background:**

Lung cancers consist of four major types that and for clinical-pathological reasons are often divided into two broad categories: small cell lung cancer (SCLC) and non-small cell lung cancer (NSCLC). All major histological types of lung cancer are associated with smoking, although the association is stronger for SCLC and squamous cell carcinoma than adenocarcinoma. To date, epidemiological studies have identified several environmental, genetic, hormonal and viral factors associated with lung cancer risk. It has been estimated that 15-25% of human cancers may have a viral etiology. The human papillomavirus (HPV) is a proven cause of most human cervical cancers, and might have a role in other malignancies including vulva, skin, oesophagus, head and neck cancer. HPV has also been speculated to have a role in the pathogenesis of lung cancer. To validate the hypothesis of HPV involvement in small cell lung cancer pathogenesis we performed a gene expression profile of transgenic mouse model of SCLC induced by HPV-16 E6/E7 oncoproteins.

**Methods:**

Gene expression profile of SCLC has been performed using Agilent whole mouse genome (4 × 44k) representing ~ 41000 genes and mouse transcripts. Samples were obtained from two HPV16-E6/E7 transgenic mouse models and from littermate's normal lung. Data analyses were performed using GeneSpring 10 and the functional classification of deregulated genes was performed using Ingenuity Pathway Analysis (Ingenuity^® ^Systems, http://www.ingenuity.com).

**Results:**

Analysis of deregulated genes induced by the expression of E6/E7 oncoproteins supports the hypothesis of a linkage between HPV infection and SCLC development. As a matter of fact, comparison of deregulated genes in our system and those in human SCLC showed that many of them are located in the Aryl Hydrocarbon Receptor Signal transduction pathway.

**Conclusions:**

In this study, the global gene expression of transgenic mouse model of SCLC induced by HPV-16 E6/E7 oncoproteins led us to identification of several genes involved in SCLC tumor development. Furthermore, our study reveled that the Aryl Hydrocarbon Receptor Signaling is the primarily affected pathway by the E6/E7 oncoproteins expression and that this pathway is also deregulated in human SCLC. Our results provide the basis for the development of new therapeutic approaches against human SCLC.

## Background

Human papillomaviruses (HPVs) are a collection of over 200 viruses that can infect humans. HPV is most often spread through skin-to-skin contact, usually sexually. Genital HPV infections are very common and are sexually transmitted. Most HPV infections occur without any symptoms and go away without any treatment over the course of a few years. However, HPVs infection sometimes persists for many years in the host, either through the establishment of latent or chronic infections, which can ultimately lead to cellular transformation [1]. It is now well-established that high-risk HPVs play a role in most cases of cervical cancer, as well as many cases of vulvar, penile, and anal cancers [2,3]. HPV 16 and 18 have been identified not only in gynecological carcinomas but also in tumors of other organs, like the upper aerodigestive tract and oropharynx especially those occurring in young, non-smoking women. Only a few of these viruses are considered the "cancer-causing" strains, most notably, HPV 16 and HPV 18 [4-6].

The possibility that HPV may play a role in the development of lung cancer was first suggested by Syrjanen in 1979 who described epithelial changes in bronchial carcinomas closely resembling those of established HPV lesions in the genital tract [7]. Since then, several studies provided evidence of HPV 16 and 18 DNA in lung cancers, but there were inconsistency in the reported prevalence of infection by HPVs in patients with lung cancer in different countries, with racial and geographic variations. In the United States, HPVs DNA is found in about 20-25% of lung cancers [8]. The most common strains found are HPV 16 and HPV 18, the same strains that are commonly found in cervical cancer. More than 90% of lung cancer in Taiwanese females is not related to cigarette smoking and 55% had HPV16/18 DNA compared with 11% of non cancer control subjects. Additionally HPV 16/18 DNA has been uniformly detected in lung tumor cells but not in the adjacent noninvolved lung tissue [9]. HPV 16/18 have been detected in the blood of women with cervical infection suggesting that HPV 16/18 can infect the lung through hematic spread from infected sites [10].

A recent review summarizes the studies conducted to establish the association between the presence of HPVs and lung cancer [11]. HPVs detection rates in lung cancer are highly variable in the different studies published from several countries, ranging from 0% to 79%. The mean incidence of HPVs in lung cancer considering all reviewed articles is 24.5%. While in Europe and in the USA the average reported incidences is 17% and 15%, respectively, the mean incidence of HPVs in Asian lung cancer is 35.7%. The authors concluded that HPV may be the second leading cause of lung cancer after cigarette smoking.

Although studies of viral-related lung cancer have been reported, the molecular mechanisms of this disease remain unclear [12-14]. Therefore, an increase in knowledge of factors promoting lung carcinogenesis, as the infection with human papillomavirus, gains in importance.

In this study we examined the gene expression profile of previously described transgenic mouse model (CK5-PAP-2303) of SCLC induced by HPV-16 E6/E7 oncoproteins [15] and compared data with those obtained from human tissue with SCLC.

The aim of our study was to identify molecular mechanisms associated to SCLC development induced by HPV 16 oncoproteins and in patients affected by SCLC to validate our "in vivo" model and the derived cell lines for the development and evaluation of new anticancer molecules.

## Methods

### RNA purification, labelling and oligonucleotides microarray hybridization

Lung tissues from 9-month-old wild-type and transgenic mice, were homogenised in Qiazol solution (Qiagen) by rotor-stator and RNA was extracted using RNeasy mini kit from Qiagen according to manufacturer's protocol. RNA samples were analyzed quantitatively and qualitatively by NanoDrop ND-1000 UV-Vis Spectrophotometer (NanoDrop Technologies, Wilmington, DE) and by Bioanalyzer (Agilent Technologies, Palo Alto, CA). Only samples with R.I.N. (RNA Integrity Number) >8.0, 260/280 nm absorbance >1.8 and 260/230 absorbance >2, were used for RNA labelling. Total RNA from lung tumor and controls, was amplified in the presence of cyanine-3/cyanine-5 labelled CTP using Agilent low RNA Input Fluorescent Linear Amplification kit (Agilent Technologies, Palo Alto, CA) according to manufacturer's protocol. After labelling, targets were purified using Qiagen's RNeasy mini spin column to remove unincorporated dye-labelled nucleotides. The quality of labelled targets was determined by calculating the amount of cDNA produced, the pmoles of dye incorporated and the frequency of incorporation by NanoDrop. Equal amounts of cRNAs (825 ng) from control (labelled with Cy3) and from transgenic mouse (labelled with Cy5) were mixed together and hybridized to the microarray in a hybridization oven at 65°C for 17 hours with rotation at 10 rpm. Gene expression profile of transgenic SCLC has been performed using Agilent whole mouse genome (4 × 44k) representing ~ 41000 genes and mouse transcripts. Samples were obtained from two HPV16-E6/E7 transgenic mice and from 2 littermate's normal lung. For each sample were performed the technical replicates.

After hybridization slides were washed with Gene Expression Wash buffer 1 for 1 minute at room temperature and Gene Expression Wash buffer 2 for 1 minute at 37°C. Finally to dry the slides and prevent ozone degradation arrays were treated with the Stabilization and Drying Solution (Agilent Technologies, Palo Alto, CA) for 30 seconds at room temperature. After wash the slides were scanned with the Agilent's dual-laser microarray scanner (G2565AA) and image data were processed using Agilent Feature extraction software (FE) (Agilent Technologies). This software calculates log ratios and p-values for valid features on each array and provides a confidence measure of gene differential expression performing outlier removal and background subtraction. Furthermore, FE filters features that are not positive and significant respect to background and/or saturated. FE was also used to perform linear and LOWESS dye normalization to correct dye bias.

### Microarray data analysis

The raw data and associated sample information were loaded and processed by GeneSpring^® ^10 (Agilent Technologies). Statistical analysis was performed using background-corrected mean signal intensities from each dye channel. Microarray data were normalized using intensity-dependent global normalization (LOWESS). Differentially expressed RNAs were identified using a filtering by the Benjamini and Hochberg False Discovery Rate (p-Value < 0.05) to minimize selection of false positives. Of the significantly differentially expressed RNA, only those with greater than 2-fold increase or 2-fold decrease in expression compared to the controls were used for further analysis. All microarray data presented in this manuscript are in accordance with MIAME guidelines and have been deposited in the NCBI GEO database (The Accession Number it is available by referees).

Functional and network analyses of statistically significant gene expression changes were performed using Ingenuity Pathways Analysis (IPA) 8.0 (Ingenuity^® ^Systems, http://www.ingenuity.com). Analysis considered all genes from the data set that met the 2-fold (p-value < 0.05) change cut-off and that were associated with biological functions in the Ingenuity Pathways Knowledge Base. For all analyses, Fisher's exact test was used to determine the probability that each biological function assigned to the genes within each data set was due to chance alone.

### Histopathology

Transgenic and control lungs were removed, washed in PBS and fixed with 4% buffered formaldehyde. Samples were processed and paraffin-embedded. Sections were stained with hematoxylin/eosin and observed with a Zeiss light microscope.

### Semiquantitative reverse transcriptase-PCR

Semiquantitative reverse transcriptase-PCR (RT-PCR) was done essentially as previously described [16]. RNA (2 μg/reaction) was used to generate cDNA and the appropriate individual pairs of oligonucleotides (40 pmol/reaction) for the test genes were used to amplify DNA from the cDNA. Semiquantitative PCR was done by using 100 μL reaction volumes and taking 33 μL aliquots at 25, 30, and 35 cycles. The expression of 18 S mRNA, which is ubiquitously expressed, was determined for each RNA sample to control for variations in RNA quantity. Ten microliters of each reaction were electrophoresed in a 1% agarose gel containing ethidium bromide. The gel was then developed using the GelDoc XR system (Bio-Rad) and quantified using Quantity One (Bio-Rad).

The Bioethics Committee of the University of Siena approved all the experiments conducted on live animals. All the experiments were performed in accordance with guidelines and regulations.

## Results

### Gene expression profiles

**Figure 1:**
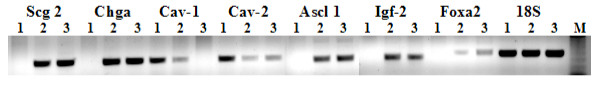
**Confirmation of microarray data**. RT-PCR was done using total RNA from wild-type mouse lung (line 1), transgenic mouse lung (line 2) and PPAP9 cells (line 3). Scg2: secretogranin 2, Chga: chromogranin, Cav-1: caveolin1, Cav-2: caveolin 2, Ascl1: achete-scute complex homologue 1, Igf-2: insulin-like growth factor 2, FoxA2: forkhead box A2, A, 18S: 18 S ribosomal RNA M: marker.

To identify mechanisms associated with SCLC development and its neuroendocrine differentiation associated to E6/E7 oncoproteins expression, we analyzed the gene expression profile of transgenic lung tumor through microarrays. Experiments were performed on lung samples from two different transgenic animals compared to normal lung tissue. To identify the differentially expressed genes, using the criteria described in Materials and Methods for Microarray data analysis, we found 5307 significantly deregulated genes. Among these 2242 genes were up-regulated and 3065 were downregulated. Up and down regulated genes are reported in the additional file 1. For each gene the probe ID, fold change, p-value, gene symbol, Gene Bank and description are reported. Interestingly, among all the genes deregulated by the E6/E7 co-expression, 116 genes are associated to neurogenesis. The list of these genes is reported in additional file 2. These results support the hypothesis of a possible role of E6 and E7 in the induction of neuroendocrine differentiation of SCLC. To confirm gene array analysis data and to validate some genes involved in this process, we performed semiquantitative RT-PCR using RNA purified from transgenic lung tumour, from littermate normal lung and from the PPAP9 cell line, established from the transgenic lung tumour [16]. As shown in Figure 1 Ascl1, Igf2, Scg2, Chga and Foxa2, considered reliable markers of neuroendocrine differentiation, are up-regulated in tissue and cells from tumour induced by E6/E7 compared to normal lung. Furthermore Cav1 and Cav2 are down regulated, according to previously published results showing a tumor suppressor activity of Caveolin-1 and its down-regulation during lung cancer development [17]. The relative direction of expression was the same for both the RT-PCR and microarray results. The primers used for RT-PCR are reported in additional file 3.

**Figure 2:**
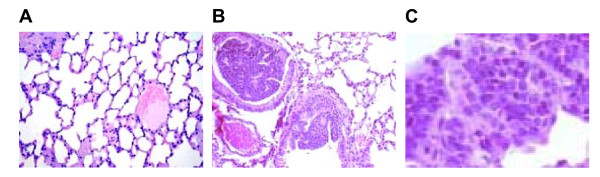
**Normal lung tissue and transgenic lung tumor histology**. (A) Normal lung; original magnification 150×; (B) Transgenic SCLC; original magnification 150×; (C) Transgenic SCLC; original magnification 400×.

The transgenic mice develop brochiogenic lung cancer around 6 months of age, and the E6/E7 genes were efficiently expressed in pre-neoplastic and neoplastic cells. The transgenic mice lung tumors showed a progression from in situ to invasive carcinoma and in a minor percentage brain, liver and pancreas metastases were observed. Inactivation of p53 and pRB occurs in the majority of neuroendocrine lung carcinomas in humans and these observations strongly suggest that E6/E7 expression most probably causes lung cancer in our transgenic model, through the inactivation of p53 and pRB. The transgenic lung carcinoma progress to multiple and bilateral tumors with histopathology and immunophenotype closely mirroring human SCLC constituted by small cells with a very high nucleus/cytoplasm ratio. Histological examination of mice lungs showed the occurrence of multiple dysplastic foci of clustering small cells in the bronchial and bronchiolar mucosa as shown in Figure 2. Furthermore intrapulmonary tumors aggressively invade lung parenchyma and vessels and readily metastasized to extra pulmonary sites, again very similar to human SCLC. Moreover a subcutaneous injection of two murine cell lines (PPAP9 and PPAP10), established from the transgenic SCLC, form primary tumors as well metastasis typical of the pattern seen in human SCLC patients [16]. The histological and biological properties of our model are overlapping to those of two other described murine SCLC models, obtained using different experimental approaches [18,19].

**Figure 3:**
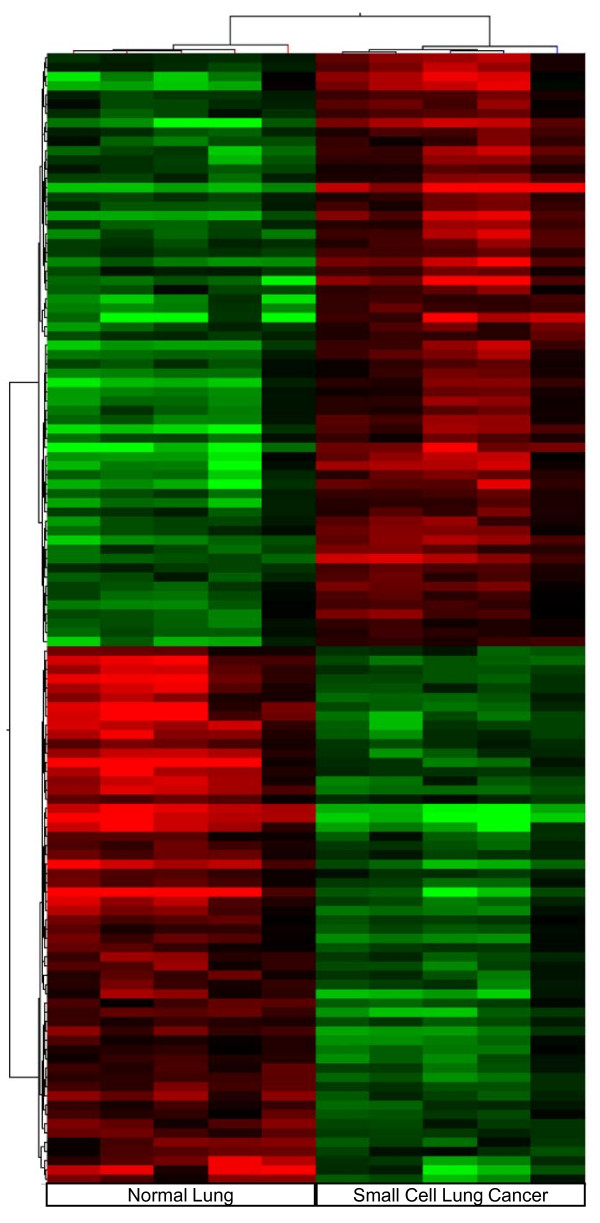
**Human SCLC hierarchical clustering of the significantly deregulated genes**. Analysis show human normal control lung tissues and SCLC samples. Up-regulated genes are shown in red, down-regulated genes are shown in green and black bars indicate not significantly changed genes.

**Figure 4:**
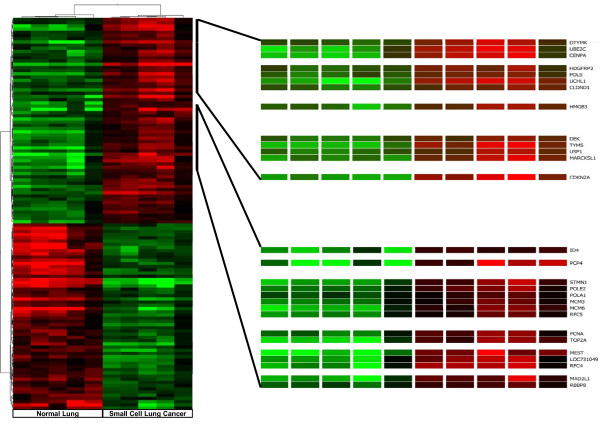
**Expansion of the significantly up-regulated genes human SCLC hierarchical clustering**. The expansion highlights the common up-regulated genes in human SCLC and in SCLC transgenic mouse induced by E6/E7 oncoproteins.

**Figure 5:**
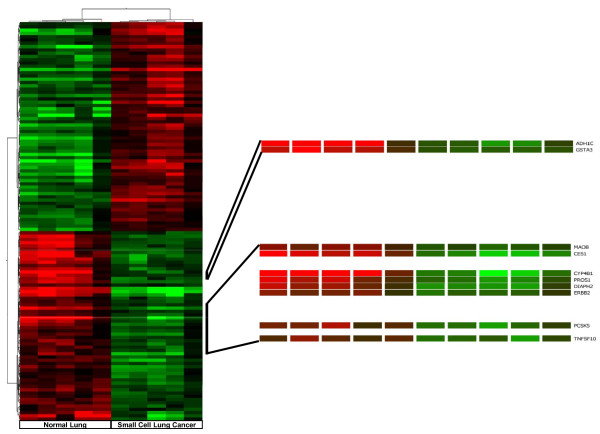
**Expansion of the significantly down-regulated genes human SCLC hierarchical clustering**. The expansion highlights the common down-regulated genes in human SCLC and in SCLC transgenic mouse induced by E6/E7 oncoproteins.

Human SCLC metastasizes early and widely and usually it is not treatable by surgery making tissue retrieval particularly difficult. Therefore the results obtained in our experimental system were compared with those available for human SCLC in Gene Expression Omnibus (GEO), a public functional genomics data repository. We imported data files from GSE6044, selecting five sets derived from human normal lung, (GSM140185-GSM140189), and five sets from human SCLC, (GSM140176-GSM140180) [20] and analyzed data sets with GeneSpring 10. Of the 8793 genes examined, 561 were differentially expressed to a significant degree (ANOVA, p < 0.05). Among these, 289 genes were up-regulated and 272 were down-regulated. Genes are listed in the additional file 4 reporting for each gene, probe ID, the fold change, p-value, gene symbol, Gene Bank and description. The significant difference in the number of deregulated genes from the two analyses is associated with the different number of genes present in the arrays, 44000 for the Agilent system and 8793 for the Affymetrix platform. Hierarchical clustering of the human differentially expressed genes according to their expression patterns is reported in Figure 3. Genes up-regulated in human SCLC are shown in red, down-regulated genes are shown in green, while black bars indicate genes that are expressed at similar levels in both. To highlight the molecular mechanisms common to tumor induced by E6/E7 oncoproteins and human SCLC, we compared the results obtained in the two systems. We identified 130 up- and 72-down regulated genes common to human SCLC and the E6/E7 induced lung tumour. The list of genes is reported in the additional file 5 showing for each gene, description, gene symbol, family name, probe ID for transgenic mouse (Agilent), probe ID for human SCLC (Affymetrix), the fold change and relative p-values. To underline similarities among samples and among genes, we overlaid the unsupervised two-dimensional hierarchical clustering, obtained from expression profile of human SCLC with results obtained from the transgenic tumour. Figure 4 and 5 highlight the expansions of the hierarchical tree containing commonly deregulated genes.

### Gene network and pathway analysis

We then used Ingenuity Pathways Analysis to highlight the cellular functions and signaling pathways affected by the E6/E7 co-expression.

The analysis of 5307 differentially expressed genes of SCLC transgenic mouse showed that the molecular and cellular functions primarily affected by the E6/E7 coexpression are associated to cellular development, cell cycle, cellular growth and proliferation. Interestingly, the analysis of 561 genes differentially expressed in human SCLC showed the involvement of the same molecular and cellular functions. Furthermore, the top five canonical pathways affected by the E6/E7 expression based on their significance, p-value < 0.01, included the Aryl Hydrocarbon Receptor Signaling, role of BRCA1 in DNA Damage Response, LPS/IL-1 Mediated Inhibition of RXR Function, role of CHK Proteins in Cell Cycle Checkpoint Control and Pyrimidine Metabolism.

**Figure 6:**
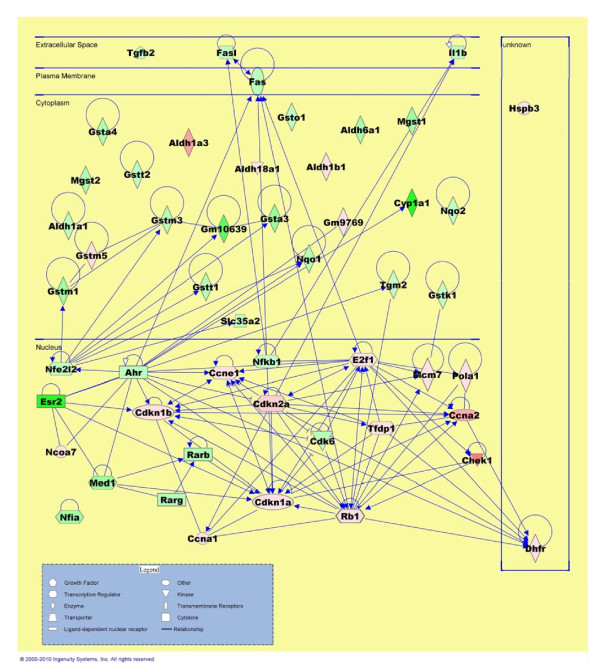
**IPA pathway graphical representation of Aryl Hydrocarbon Receptor Signaling**. 51 deregulated genes are represented out of 154. Gene products are positioned according to sub cellular localization. Only direct connections (i.e., direct physical contact between two molecules) among the individual gene products are shown for clarity of presentation; lines indicate protein-protein binding interactions, and arrows refer to "acts on" interactions such as proteolysis, expression, and protein-protein interactions. Genes up regulated are shown in red, down-regulated genes are shown in green.

Canonical pathway analysis of transgenic SCLC revealed the Aryl Hydrocarbon Receptor Signaling as the most significant signaling pathway modulated by E6/E7 expression (p-value 1.89 × 10^-7^). Fifty-one genes in this pathway were deregulated with 20 of them up-regulated and 31 down-regulated. We used these genes to assemble the pathway depicted in Figure 6. Fifty-one deregulated genes out of one hundred fifty-four total genes that map the canonical pathway Aryl Hydrocarbon Receptor Signaling are positioned according to subcellular localization. The genes in this pathway have ascribed not only to detoxification mechanism, but also to functions such as cell cycle progression, cancer and cell proliferation. Cyclin dependent kinase inhibitor 2A (CDKN2A) occupies a focal position in this pathway; up-regulation of this gene has been previously suggested to be a specific marker for dysplastic and neoplastic epithelial cells of the cervix uteri [21].

In addition, canonical pathways were also evaluated within the human SCLC. The top five canonical pathways modulated in human tumour, based on their significance, pvalue < 0.01, included the Metabolism of Xenobiotics by Cytochrome P450, Pyrimidine Metabolism, Bile Acid Biosynthesis, Aryl Hydrocarbon Receptor Signaling and Mitotic Roles of Polo-Like Kinase. Evaluation of the results obtained in the two systems showed deregulation of the same pathways in human SCLC and in that induced experimentally by the E6/E7 oncoproteins of HPV16.

**Figure 7:**
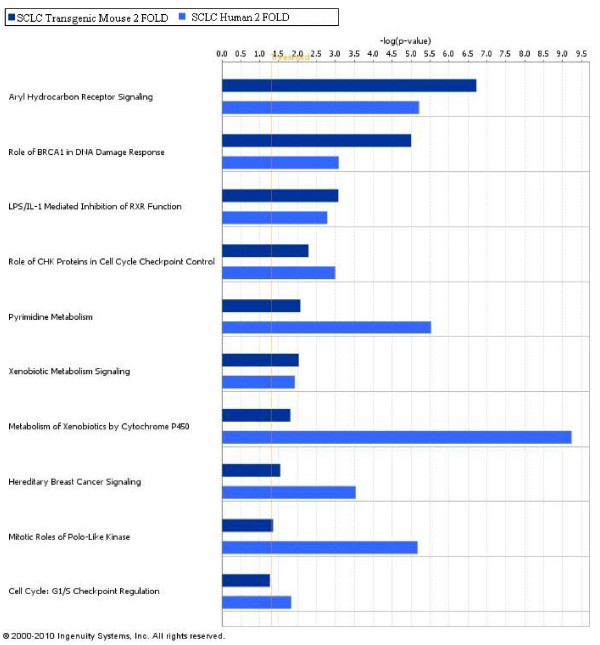
**Comparison analysis of most significant pathways in human SCLC and in SCLC induced by E6/E7**. The comparison of top ten canonical pathways identified by IPA in human SCLC and in transgenic mouse emphasizes the common differential regulation in the tumor development.

To further highlight the similarity of the two systems, the comparison analysis is shown in Figure 7 where the first ten canonical pathways based on their significance (p-value < 0.01) are reported.

## Discussion

Human papillomaviruses (HPVs) are small non-enveloped DNA viruses that infect squamous epithelial cells. HPVs give rise to a large spectrum of epithelial lesions, mainly benign hyperplasia with low malignant potential. A subgroup of HPVs, the "high-risk" HPV, is associated with precancerous and cancerous lesions. A small fraction of people infected with high-risk HPV will develop cancers that usually arise many years after the initial infection [1].

The high-risk HPV E6 and E7 joint expression is necessary and sufficient for the immortalization of primary human keratinocytes in vitro [22]. In squamous cell carcinomas of the head and neck (HNSCC), the E6 and E7 oncoproteins function through multiple interactions with two cardinal cellular regulators of cell cycle, the tumor suppressor protein 53 (p53) and the retinoblastoma gene product (pRb), respectively [23,24].

The E6 protein inactivates p53 by complex formation or triggering its ubiquitinmediated degradation. The E7 protein inactivates pRb by binding the transcription factor E2F when pRb is unphosphorylated. Both, pRb phosphorylated by cyclindependent kinases and pRb bound by E7 release the E2F transcription factor, subsequently leading to progression of the cell into the S-phase [14]. Furthermore, E7 binds to inhibitors of cyclin-dependent kinases (p16, p21), increasing the level of phosphorylated pRb. In this way, HPV 16 oncoproteins induce the failure of cell cycle regulation with lack of p53 mutations, a common feature of many human cancers [25].

HPV 16/18 are known to cause cervical cancer and has been suggested to cause vulvar, vaginal and penile cancers as well anal cancers [26]. Several laboratories have demonstrated that HPV DNA could exist in peripheral blood mononuclear cells (PBMCs) of patients with genital HPV 16 infection and with cervical cancer. HPV16 genome exists in PBMCs of pediatric HIV patients who acquired HIV infection via transfusion and in "healthy" blood donors, suggesting a potential transmission via the bloodstream [27]. Recent studies suggest that HPV infection may also play a role in the development of oral cancer [28], esophageal cancer [29] and colorectal cancer [30]. Furthermore the association between the presence of HPV 16 and the development of head and neck cancer has been recently established [31]. The possible involvement of HPV in bronchial squamous cell lesions was first suggested in 1979 by Syrjanen who described epithelial changes in bronchial carcinomas closely resembling those of established HPV lesions in the genital tract [7]. HPV 16/18 are established causative role in upper airway cancer. Whereas HPV 16/18 have been detected in the blood of women with cervical infection, it has been suggested that HPVs can infect the lung through hematogenous spread from infected sites [10]. Variability in reported number of HPV-positive lung cancer may be explained by several factors, such as environmental variables, high-risk behavior, genetic susceptibility, and methodologic approaches with varying sensitivity and specificity for HPVs identification [32]. The reasons for having false-negative detection of HPVs are the use of inappropriate primers or loss of the HPV L1 and E2 genes during integration.

The aim of this study was to identify the molecular mechanisms commonly deregulated in SCLC induced by viral oncoproteins and in patients with SCLC. Therefore, we examined the gene expression profiles of transgenic mouse model induced by HPV-16 E6/E7 oncoproteins and compared data with those obtained from human tissues with SCLC. The analysis highlights that several molecular mechanisms are common to tumor induced by E6/E7 oncoproteins and human SCLC. In particular, the Aryl Hydrocarbon receptor signaling is the predominant pathway deregulated in both systems. The aryl hydrocarbon receptor (AHR) is a cytosolic ligand-activated transcription factor that mediates many toxic and carcinogenic effects in animals and in humans [33]. The mechanism of action of aryl receptor signaling has been extensively studied as a function of exposure to TCDD. Among the results tissue remodelling has been associated with its deregulation. In the absence of such induction, other studies have highlighted the aryl receptor signaling involvement in other pathophysiological conditions. Outside its well-characterized role, the AHR also functions as a modulator of cellular signaling pathways.

AHR can trigger signal transduction pathways involved in proliferation, differentiation or apoptosis by mechanisms that may be ligand mediated or completely ligand independent [34]. Several published accounts point to a role for AHR in cell cycle control, although the precise mechanism is still unclear. Two different signaling pathways contribute to the role of AHR in cell cycle regulation. AHR promotes apoptosis, repressing TGFβ1 expression by accelerating TGFβ1 mRNA degradation [35]. In addition, fibroblasts from AHR-knockout mice overproduce TGFβ1 causing low proliferation rates and increased apoptosis [36]. The aryl hydrocarbon receptor is a member of transcription factor controlling a variety of developmental and physiological events including not only drug metabolism and hence the xenobiotic detoxification but also neurogenesis [33].

Thus, depending on the cellular environment, Ahr could be considered a pro-proliferative gene in some cases and an anti-proliferative gene in others. The AHR ligand-mediated repression of previously active genes that might have little connection with detoxification pathways and concomitant induction of previously silent genes are likely to affect cellular homeostasis.

Protein interaction between AHR and the retinoblastoma protein, well know to be inactivated in the SCLC (Rb/E2F axis) repress S phase gene expression and prevent entry of cells in the S phase [37]. Furthermore, other members of aryl hydrocarbon receptor signaling, the inhibitors of cyclin-dependent kinases (p16 and p21), have been demonstrated to bind E7 increasing the level of pRb phosphorylation. In our paper we provide evidences of connections between different signal transduction pathways that cross-talk with the AHR suggesting a role of aryl hydrocarbon receptor signaling deregulation in the SCLC development. We don't know if the deregulation of many members of this pathway is the cause or the effect of SCLC development and the exact molecular mechanisms by which AHR exerts its effects remain to be further analyzed.

Finally additional researches are needed to establish definitive evidence of HPV as an etiological factor of human SCLC and further proof will be provided by the impact on the lung cancer incidence of HPV-directed vaccine meant to prevent cervical cancer.

## Conclusion

Using a genome-wide expression analysis of a transgenic mouse model of SCLC induced by HPV-16 E6/E7 oncoproteins we tested the hypothesis of a correlation between HPV infection and lung cancer development. The analysis led to the identification of several genes commonly deregulated in the murine model and in human SCLC. Although we do not provide definitive proof of direct connection between HPV infection and SCLC development, our results support the hypothesis of HPV as a risk factor and/or cofactor in the SCLC development. Furthermore, the study reveled that the Aryl Hydrocarbon Receptor Signaling is the primarily affected pathway by the E6/E7 oncoproteins expression and that this pathway is also deregulated in human SCLC. Finally, the identification of molecular mechanisms associated to SCLC development induced by HPV 16 oncoproteins and in patients affected by SCLC validate our "in vivo" model and the derived cell line PPAP9 for the designing and testing new therapeutic strategies against human SCLC.

## Competing interests

The authors declare that they have no competing interests.

## Authors' contributions

TB performed the Microarray, RT-PCR experiments and drafted the manuscript. LC participated in mouse colony maintenance, organ collection and carried out histopathology analysis. MR contributed to study conception. RM designed and coordinate the study, carried out microarray analysis (GeneSpring and IPA software), and wrote the manuscript. All authors read and approved the final manuscript.

## Supplementary Material

Additional file 1 **Significantly deregulated genes in SCLC induced by E6/E7 oncoproteins**. Click here for file

Additional file 2 **Deregulated genes by the E6/E7 co-expression associated to neurogenesis**. Click here for file

Additional file 3 **RT-PCR primers used to test the expression of several selected genes to validate the microarray data and neurogenesis differentiation**. Click here for file

Additional file 4 **Human SCLC Deregulated Genes**. Click here for file

Additional file 5 **Common deregulated genes in human SCLC and in E6/E7 induced lung tumor**. Click here for file
