# Supplementary material for: Involvement of aryl hydrocarbon receptor signaling in the development of small cell lung cancer induced by HPV E6/E7 oncoproteins
Source: J Transl Med. 2011 Jan 4;9:2. doi: 10.1186/1479-5876-9-2 (PMC3022727; doi:10.1186/1479-5876-9-2)
Supplement: Additional file 3 — RT-PCR primers used to test the expression of several selected genes to validate the microarray data and neurogenesis differentiation. [file 1479-5876-9-2-S3.DOC]

**Additional File 3 Additional Table 3: RT-PCR primers used to test the**

**expression of several selected genes**

| Gene | Primer Forward | Primer Reverse |
| --- | --- | --- |
| 18S | 5’ AGTTGGAGCGATTTGTC 3’ | 5’ AGGGCCTCAAACCATCC 3’ |
| Ascl1 | 5’ GTTGGTCAACCTGGGTTTTG 3’ | 5’ TGACGTCGTTGTCAAGAAACA 3’ |
| Chga | 5’ TAACCAAGAGTCCCCCATGA 3’ | 5’ TTCCTCCTGCTGAGAGTGCT 3’ |
| Foxa2 | 5’ CAGTAGCGGAGGCAAGAAGA 3’ | 5’ CCTTGAGGTCCATTTTGTGG 3’ |
| Igf2 | 5’ GTTCAGAGAGGCCAAACGTC 3’ | 5’ GGGTGTCAATTGGGTTGTTT 3’ |
| Scg2 | 5’ ATGACATCCCAGAGGCTGAC 3’ | 5’ GGACTGGGCACTCTCTTCAG 3’ |
| Cav1 | 5’ CCTTCTCGCATCCACTCTTT 3’ | 5’ AGGCAGTTGAGGTTGTTGGT 3’ |
| Cav2 | 5’ TCCGTGCAGACAATATGGAA 3’ | 5’ TGAGGAAAGTTGCCCTTTGAC 3’ |
